# Red Marrow Absorbed Dose Calculation in Thyroid Cancer Patient Using a Simplified Excel Spreadsheet

**DOI:** 10.4274/mirt.galenos.2020.71473

**Published:** 2020-10-19

**Authors:** Rangsee Songprakhon, Krisana Roysri, Putthiporn Charoenphun, Krisanat Chuamsaamarkkee

**Affiliations:** 1 Surin Hospital, Clinic of Radiology, Division of Nuclear Medicine, Surin, Thailand; 2 Mahidol University Faculty of Medicine Ramathibodi Hospital, Department of Diagnostic and Therapeutic Radiology, Bangkok, Thailand

**Keywords:** Red marrow absorbed dose, image-based dosimetry, radioiodine therapy, internal dosimetry

## Abstract

**Objectives:**

Absorbed dose to red marrow (D_rm_) can be calculated using blood dosimetry. However, this method is laborious and invasive. Therefore, image-based dosimetry is the method of choice. Nonetheless, the commercial software is expensive. The goal of this work was to develop a simplified excel spreadsheet for image-based radioiodine red marrow dosimetry.

**Methods:**

The serial whole-body images (acquired at 2^nd^, 6^th^, 24^th^, 48^th^, and 72^th^ hours) of 29 patients from the routine pretherapeutic dosimetry protocol were retrospectively reanalyzed. The commercial OLINDA/EXM image-based dosimetry software was used to calculate the whole-body time-integrated activity coefficient (TIAC_WB_) and D_rm_ [in terms of absorbed dose coefficient (d_rm_)]. For the simplified excel spreadsheet, the whole-body count was obtained from the vendor-supplied software. Then, the TIAC_WB_ was computed by a fitting time-activity curve using an Excel function. S factor was taken from other publications and scaled according to the patient-specific mass. A comparison of the TIAC_WB_ and d_rm_ from both methods was done using a non-inferiority test using a paired t-test or the Wilcoxon signed-rank test.

**Results:**

The TIAC_WB_ showed no significant difference between both methods (p=0.243). The calculated D_rm_ from a simplified Excel spreadsheet was assumed to be statistically non-inferior to the commercial OLINDA/EXM image-based dosimetry software with the non-inferiority margin of 0.02 (p<0.05).

**Conclusion:**

The dose assessment from a simplified Excel spreadsheet is feasible and relatively low cost compared to the commercial OLINDA/EXM image-based dosimetry software.

## Abstract (tr)

**Amaç:**

Kırmızı iliğin absorbe ettiği doz (D_rm_) kan dozimetresi kullanılarak hesaplanabilir. Ancak tam kan yöntemi zahmetli ve invaziftir. Bu nedenle, görüntü bazlı dozimetri tercih edilen yöntemdir. Bununla birlikte, ticari yazılım pahalıdır. Bu çalışmanın amacı, görüntü tabanlı radyoiyot kırmızı ilik dozimetrisi için basitleştirilmiş bir excel elektronik tablosu geliştirmektir.

**Yöntem:**

Yirmi dokuz hastanın rutin tedavi öncesi dozimetri protokolündeki seri tüm vücut görüntüleri (2, 6, 24, 48 ve 72. saatlerde elde edilen) retrospektif olarak yeniden analiz edilmiştir. Rutin olarak, ticari OLINDA/EXM görüntü tabanlı dozimetri yazılımı, tüm vücut zamana entegre edilmiş aktivite katsayısı (TIAC_WB_) ve D_rm_ [absorbe edilen doz katsayısı (d_rm_)] hesabı için kullanıldı. Basitleştirilmiş bir Excel elektronik tablosu için, tüm vücut sayısı satıcı tarafından sağlanan yazılımdan elde edilmiştir. Daha sonra TIAC_WB_, Excel işlevi kullanılarak oluşturulan zaman-aktivite eğrisi ile hesaplandı. S faktörü diğer yayınlardan alınarak hastaya özel kitleye göre ölçeklendirildi. İki yöntem ile elde edilen TIAC_WB_ ve d_rm_ değerlerinin karşılaştırması, bağımlı örneklem t-testi veya Wilcoxon işaretli sıralar testine dayalı bir non-inferiority test kullanılarak analiz edildi.

**Bulgular:**

TIAC_WB_ iki yöntem arasında anlamlı bir fark göstermedi (eşleştirilmiş örneklem t-testi ile p değeri 0,243). Basitleştirilmiş bir Excel elektronik tablosundan hesaplanan d_rm_ değerinin, 0,02’lik non-inferiority payı ile ticari OLINDA/EXM görüntü tabanlı dozimetri yazılımı ile hesaplanan değerden istatistiksel olarak non-inferior olduğu görülmüştür (Wilcoxon işaretli sıralar testi ile p değeri <0,05).

**Sonuç:**

Basitleştirilmiş bir Excel elektronik tablosundan doz değerlendirmesi yapılabilir ve ticari OLINDA/EXM görüntü tabanlı dozimetri yazılımına kıyasla nispeten düşük maliyetlidir.

## Introduction

The red marrow is considered as one of the critical organs in radioiodine treatment (^131^I-sodium iodide) of differentiated thyroid cancer (DTC). In radioiodine treatment, the absorbed dose to the blood as a surrogate for red marrow is often kept below 2 Gy to avoid hematological toxicity. This safety limit has been defined by blood dosimetry from blood sampling since the original work of Benua et al. (1) in 1962. Such a limit is still widely accepted, even though many new approaches have been introduced to calculate the red marrow absorbed dose such as external whole-body counting using a gamma probe and quantitative imaging using serials of whole-body scans obtained from nuclear medicine imaging modality (2,3,4).

Currently, quantitative imaging is the method of choice due to its non-invasive procedures excluding serial blood collections. Several commercial dosimetry softwares include the function to calculate the time-integrated activity coefficient (TIAC) and absorbed dose in organs using either serial planar whole-body scans or single photon emission computed tomography (SPECT) acquisitions (3,4). However, the image-based commercial dosimetry software is expensive, limiting its use in routine clinical practice. The objectives of this study were to develop a simplified excel spreadsheet for an image-based radioiodine bone marrow dosimetry and to compare the results of this spreadsheet with the commercial OLINDA/EXM image-based dosimetry software.

## Materials and Methods

### Patient Data Selection

Twenty-nine DTC patients who participated in the routine pretherapeutic dosimetry protocol between May 2017 to March 2019 for radioiodine treatment at the Surin Hospital (Surin Province, Thailand) were included in this retrospective study. Five patients were male and 24 were female. The mean age was 48.8 years (range: 19.0-76.0 years) at the time of the treatment. Ethics Committee Approval was obtained from the Ethical Review Board of the Surin Hospital with the approval number: 12/2562 and date: 9^th^ April 2019.

The inclusion criteria for this routine pretherapeutic dosimetry protocol were DTC patients who had a near-total or total thyroidectomy, with withdrawn thyroid hormone for 4-6 weeks, low iodine diet intake, and serum thyroid-stimulating hormone >30 mIU/L before administration of radioiodine.

### Dosimetry and Imaging Protocol

The pretherapeutic dosimetry protocol at the Surin Hospital was performed following European Association of Nuclear Medicine (EANM) standard operational procedures (3). Radioiodine ranging from 74 to 185 MBq was administered to patients. The whole-body data in this protocol were obtained from anterior and posterior conjugate views acquired at 2^nd^, 6^th^, 24^th^, 48^th^, and 72^th^ hours postadministration. The gamma camera used for imaging was Symbia T16 SPECT/CT (Siemens Medical Solutions USA) and equipped with parallel-hole high energy collimators, using a 10% energy window set at 364 keV. The table speed for the whole-body images was 8 cm/min and the latter were acquired using a 256x1024 matrix. These protocol settings were applied to all patients and time points.

### The Commercial OLINDA/EXM Image-based Dosimetry Software

In the pretherapeutic dosimetry protocol, the whole-body  calculations were performed using a commercial HERMES OLINDA/EXM image-based dosimetry software (HERMES Medical Solution, Stockholm, Sweden). This software is the OLINDA/EXM version 1.1.

**Figure 1:**
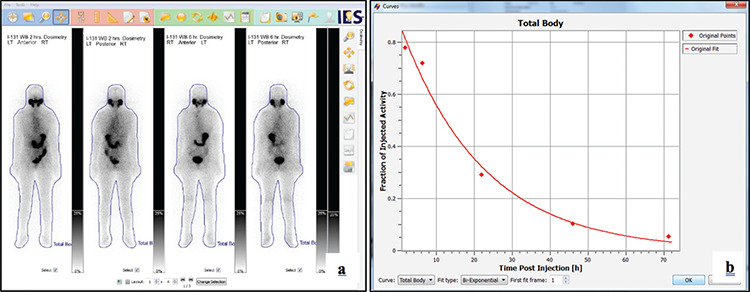
**a)** Sample screenshot of the image data and whole-body ROI of the commercial OLINDA/EXM image-based dosimetry software. **b)** The bi-exponential fit of the TAC ROI: Region of interest, TAC: Time-activity curve, OLINDA/EXM: Organ Level Internal Dose Assessmant/Expanential Medelling

In this commercial software, the whole-body region of interest (ROI) in the anterior image was automatically mirrored and copied to the posterior image. The whole-body ROIs that had been defined in one of the whole-body scans were automatically copied to all other timepoints belonging to the same patient. Examples of image data and whole-body ROI are demonstrated in Figure 1a.

In OLINDA/EXM image-based dosimetry software, the activity in the images is converted from the counts using either standard activity or equipment detection efficiency. In this work, the camera detection efficiency was investigated and used for activity determination for all patients. Then, a bi-exponential function was fitted to the data. Consequently, the whole-body time-activity curve whole-body (TAC_WB_) was generated. An example of TAC_WB_ is shown in Figure 1b.

The whole-body TIAC_WB_ (formally called residence time) is calculated from the area under TAC_WB_. The exponential extrapolation with a numerical trapezoidal integration is employed in this OLINDA/EXM image-based dosimetry software. In this software, D_rm_ was calculated in terms of the absorbed dose coefficient (d_rm_) (mGy/MBq). This commercial software uses the medical internal radiation dose schema and the Cristy and Eckerman (C&E) phantoms (5). In our work, doses were scaled using the patient-specific mass at the time of radioiodine treatment.

### A Simplified Excel Spreadsheet

**Figure 2:**
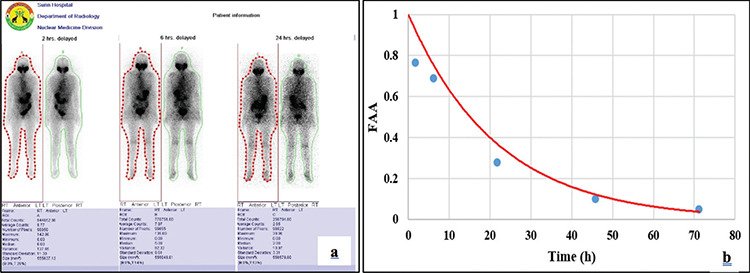
**a)** The whole-body ROI from the vendor-supplied Syngo software. **b)** The TIAC_WB_ from a simplified eExcel spreadsheet ROI: Region of interest, TIAC_WB_: Whole-body time-integrated activity coefficient

The serial whole-body images of 29 DTC patients were reanalyzed using the vendor-supplied Syngo software (Siemens Medical Solutions, USA) as illustrated in Figure 2a. Data were exported to an Excel spreadsheet (Microsoft Corp., Redmond, WA). For background correction, the average activity in the whole-body background ROIs (BKG_av_) was subtracted from the average activity in the whole-body ROI (WB_av_) and multiplied by the number of pixels in the whole-body image (N_WB_) as illustrated in equation (1).

${\mathrm{WB}}_{\mathrm{net}}=N_{\mathrm{WB}}\;\times \;({\mathrm{WB}}_{\mathrm{av}}-{\mathrm{BKG}}_{\mathrm{av}})\;\;\;\;\;\;\;\;\;\;\;\;\;\;\;\;\;\;\;\;\mathrm{equation}\;(1)$

Then, the whole-body geometric mean (WB_GM_) was calculated from the whole-body net anterior (WB_net(ant)_) and whole-body net posterior ( WB_net(post)_) as shown in equation (2).

${\mathrm{WB}}_{\mathrm{GM}}=\sqrt{{\mathrm{WB}}_{\mathrm{net}(\mathrm{ant})}\;\times \;\mathrm{WB}{\;}_{\mathrm{net}(\mathrm{post})}}\;\;\;\;\;\;\;\;\;\;\;\;\;\;\;\mathrm{equation}\;(2)$

In the excel spreadsheet, the equipment efficiency was also used to determine the activity similar to the OLINDA/EXM image-based software. Then, the whole-body activity of each time point was computed to the fraction of administered activity (FAA) using a mono-exponential function in MS Excel [as illustrated in equation (3)].

$\mathrm{FAA}(t)\;=\frac{A(t)}{A_{0}}\;A\;\times \;e^{-\lambda \times t}\;\;\;\;\;\;\;\;\;\;\;\mathrm{equation}\;(3)$

Where, FAA(t) is the fraction of administered activity (A_0_) as a function of time t and A and λ are fit constants. The TIAC_WB_ (as shown in Figure 2b) in this spreadsheet were calculated by integrating the equation (3) from zero to infinity as shown in the following equation (3, 4).

${\mathrm{TIAC}}_{\mathrm{WB}}\;\int_{0}^{\infty }=\;\mathrm{FAA}(t)\mathrm{dt}\;\;\;\;\;\;\;\;\;\;\;\mathrm{equation}\;(4)$

As recommended in the EANM guideline for bone marrow and whole-body dosimetry in radioiodine therapy for thyroid cancer, the contributors to D_rm_ were the activity in the extracellular fluid (ECF) (D_rm←ECF_) and the remainder of the body (RoB) (D_rm←_RoB) as illustrated in equation (4,5).

$\mathrm{Drm}=\;D_{\mathrm{rm}\leftarrow \mathrm{ECF}}\;+\;D_{\mathrm{rm}\leftarrow \mathrm{RoB}}\;\;\;\;\;\;\;\;\;\;\;\mathrm{equation}\;(5)$

The contribution from the activity in the ECF is also called the blood method and assumes that the activity distribution in ECF is equal to the activity distribution in the plasma. However, the completed blood method is laborious, invasive, and resource consuming. Many groups have published using this method to avoid blood sampling (2,6,7,8).

In this spreadsheet, the method introduced by Thomas et al. (2) was used to estimate the blood TIAC (TIAC_blood_) assuming that 14% of the TIAC_WB_ can be attributed to blood as shown in equation (6). Thereby, the D_rm←ECF_can be calculated by the activity concentration in blood and the red marrow ECF fraction (RMECFF) as shown in equation (7). The RMECFF was 0.19, based on Sgouros studied using a theoretical investigation of radiolabeled monoclonal antibodies (9). For D_rm←ECF_, the patient-specific bone marrow mass is not necessary as it is canceled out when the S value is scaled (2). For the S value for red marrow to red marrow, the C&E phantoms were also used (10).

${\mathrm{TIAC}}_{\mathrm{blood}}=\;0.14\;\times \;{\mathrm{TIAC}}_{\mathrm{WB}}\;\;\;\;\;\;\;\;\;\;\;\mathrm{equation}\;(6)\;D_{\mathrm{rm}\leftarrow \mathrm{ECF}}\;=\;A_{0}\;\times \;0.19\;\times \;m_{\mathrm{rm},\mathrm{phantom}}\;\times \;S_{\mathrm{rm}\leftarrow \mathrm{rm},\mathrm{phantom}}\;\;\;\;\;\;\;\;\;\;\;\mathrm{equation}\;(7)$

The remainder of the body TIAC (TIAC_RoB_) can be calculated as the difference between the TIAC_WB_ and the other source organs which is only in this case.  in this spreadsheet was calculated by subtracting the TIAC_blood_ from the TIAC_WB_ as shown in equation (8). S value for the remainder of the body to red marrow was taken from the C&E phantoms (S_rm__←__RoB, phantom_). In this case, there were only two source organs. The patient-specific S value for the remainder of the body to red marrow (S_rm__←__RoB, patient_) was calculated using a linear scaling as recommended in the EANM guideline [illustrated in equation (9)] (4).

$D_{\mathrm{rm}\leftarrow \mathrm{RoB}}=[A_{0}\times ({\mathrm{TIAC}}_{\mathrm{WB}}-{\mathrm{TIAC}}_{\mathrm{blood}})]\times \;S_{\mathrm{rm}\leftarrow \mathrm{RoB},\;\mathrm{patient}}\;\;\;\;\;\;\;\;\;\;\mathrm{equation}\;(8)\;S_{\mathrm{rm}\leftarrow \mathrm{RoB},\mathrm{patient}}\;=[S_{\mathrm{rm}\leftarrow \mathrm{WB},\mathrm{phantom}}\;.\;\frac{m_{\mathrm{WB},\mathrm{phantom}}}{m_{\mathrm{WB}},\;\mathrm{phantom}-m_{\mathrm{RM},\;\mathrm{phantom}}}\;-\;S_{\mathrm{rm}\leftarrow \mathrm{rm},\mathrm{phantom}}\;.\;\frac{m_{\mathrm{rm},\mathrm{phantom}}}{m_{\mathrm{WB},\;\mathrm{phantom}-}m_{\mathrm{rm},\;\mathrm{phantom}}}\;].\;\frac{m_{\mathrm{WB},\mathrm{phantom}}}{m_{\mathrm{WB},\;\mathrm{patient}}}\;\;\;\;\;\;\;\;\;\mathrm{equation}\;(9)$

Where, m_rm,phantom_,m_WB,phantom_ an dm_WB,patient_ are the red marrow mass, the whole-body mass of the C&E phantoms, and the whole-body mass of the patient respectively (10).

In the same manner, *d_rm_* was computed from the D_rm_ normalized by administered activity A_0_. The unit of d_rm_ is mGy/Mbq.

### Statistical Analysis

SPSS version 20 (IBM Inc., NY, USA) was used for statistical analysis. The TIAC_WB_ and the D_rm_ calculated from the commercial OLINDA/EXM image-based dosimetry software and a simplified excel spreadsheet were expressed as mean ± standard deviation (SD). A comparison of the TIAC_WB_ and the d_rm_ from both methods was statistically assessed using a non-inferiority test based on the paired t-test when normality was assumed or the Wilcoxon signed-rank test for the case of non-normality (11). Pearson’s correlation was also used to study the correlation between both methods (12).

## Results

**Figure 3:**
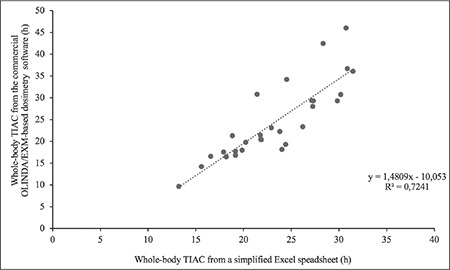
Correlation of the TIAC_WB_ calculated from a simplified Excel excel spreadsheet and the commercial OLINDA/EXM image-based dosimetry software TIAC_WB_: Whole-body time-integrated activity coefficient

This retrospective study included 29 patients who participated in the pretherapeutic dosimetry protocol for radioiodine therapy. In this work, the TIAC_WB_ and the  d_rm_ were reanalyzed using a simplified excel spreadsheet. The mean (± SD) and range (minimum-maximum) of the TIAC_WB_ and the d_rm_ are summarized in Table 1. The calculated  TIAC_WB_ showed no statistically significant difference between the two methods (p value 0.243 using a two-sided paired t-test because normality was assumed). The Pearson correlation coefficient was 0.851 (p value <0.001) (Figure 3).

**Figure 4:**
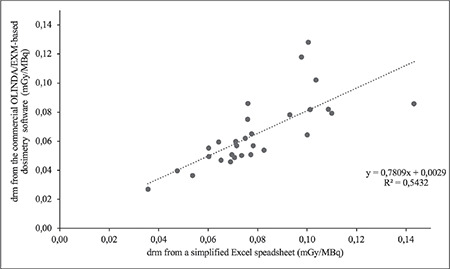
Correlation of the d_rm_ calculated from a simplified Excel excel spreadsheet and the commercial OLINDA/EXM image-based dosimetry software d_rm_: Absorbed dose coefficient

The calculated d_rm_ from the commercial OLINDA/EXM image-based dosimetry software was 0.0653±0.0233 mGy/MBq (range: 0.0268 to 0.1280 mGy/MBq). After reanalysis with a simplified excel spreadsheet, the mean (± SD) of the d_rm_ was 0.0798±0.0220 mGy/MBq (range: 0.0356 to 0.1433 mGy/MBq). For D_rm_, the statistical results showed that the calculated d_rm_ from a simplified excel spreadsheet was statistically non-inferior to that from the commercial OLINDA/EXM image-based dosimetry software with a non-inferiority margin of 0.02 (p value <0.05 using the Wilcoxon signed-rank test because non-normality was assumed). The non-inferiority margin of 0.02 was set based on SD of d_rm_ from the commercial OLINDA/EXM image-based dosimetry software that was used in the pretherapeutic dosimetry protocol. Pearson’s correlation coefficient was 0.737 (p value <0.001) as illustrated in Figure 4.

## Discussion

Image-based red marrow dosimetry calculation in radioiodine therapy is performed to maximize the radiation dose to remnant thyroid or metastasis CT while considering the patient’s safety by minimizing bone marrow toxicity. The primary parameter requested for internal dosimetry is often the TIAC (13). In this study, the calculated from a simplified excel spreadsheet was slightly shorter than that using the commercial OLINDA/EXM image-based dosimetry software. The mean percentage difference in TIAC_WB_ between both methods was 0.41%. Many factors affect TIAC estimation such as counts-to-activity conversion method, ROI delineation, background correction, and method of fit and integration of TAC.

In this study, the counts-to-activity conversion was similar using equipment efficiency for both methods. For ROI delineations, there were drastic differences between the commercial OLINDA/EXM image-based dosimetry software and a simplified excel spreadsheet. As regards illustration, the commercial OLINDA/EXM image-based dosimetry software has an advanced option to draw and automatically copy ROIs from the initial whole-body to all the other images belonging to the same patient. In contrast, the ROI in a simplified Excel spreadsheet was manually drawn using the vendor-supplied software. Many studies have reported that ROI delineation was one of the critical uncertainty factors for dose calculation in nuclear medicine (14,15,16). For the fit and integration method, a simplified excel spreadsheet was used; the mono-exponential fit (also called single exponential function) in MS Excel. The commercial OLINDA/EXM image-based dosimetry software was fitted using a bi-exponential fit function. According to the EANM guideline for pretherapeutic dosimetry in DTC, the bi-exponential fitting is suggested to determine the  (3). Many simplified approaches have been developed and illustrate that the simple mono-exponential could be used in routine practice (17,18). However, errors of the mono-exponential might be higher since such a fitting does not reflect realistic kinetics in the uptake and long-term retention phase of radioiodine kinetics (17). The most desirable next step is to include the bi-exponential fit in our excel spreadsheet to improve the accuracy of TIAC. Comparison of the  presented in this work with other publications is tabulated in Table 2.

It was found that most publications reported a large SD in the TIAC_WB_, like our findings. The possible explanations for high SD for TIAC_WB_ in radioiodine treatment might be related to the disease characteristic of ablation in DTC patients such as metastasis, iodine intake/uptake, hormonal level, age of the treated patient.

As previously explained, the d_rm_ is the ratio between the bone marrow absorbed dose and administered activity. In this study, the difference in the d_rm_ from a simplified excel spreadsheet and the commercial OLINDA/EXM image-based dosimetry software was -26.98%. Many factors affect d_rm_, including the phantom and S factor. In our study, both methods used the C&E phantoms, but different versions. The simplified excel spreadsheet used the C&E phantoms from the study of Stabin et al. (19) in 1995 whereas, the commercial OLINDA/EXM image-based dosimetry software used the newer version from the study of Stabin et al. (5) in 2003. The latter version used the bone marrow specific absorbed fraction from the EGS4 Monte Carlo code.

Statistical comparison of the d_rm_ from both methods was analyzed using a non-inferiority test. Generally, this test is used to assess that a new drug or new treatment is not worse than the main comparator drug or a reference treatment by more than a non-inferiority margin (11,20). In this study, the non-inferiority margin of 0.02 was set. This value was based on the SD of d_rm_from the commercial OLINDA/EXM image-based dosimetry software and other published works (as illustrated in Table 3). The non-inferiority test showed that a simplified Excel spreadsheet software was no worse than the commercial OLINDA/EXM image-based dosimetry software at the non-inferiority margin.

For comparison, the mean values of d_rm_ calculated from both methods were compared with the values reported by other groups as demonstrated in Table 3.

In this study, the mean and SD of d_rm_ from the simplified excel spreadsheet was 0.0798±0.0220 mGy/MBq. Traino et al. (21) and Miranti et al. (22) calculated the d_rm_ using the reference data from the RADAR website with an estimated  d_rm_ of 0.0739±0.0217 mGy/MBq and 0.0845±0.0385 mGy/MBq, respectively. Willegaignon et al.(23) also computed the d_rm_ from whole-body images using the OLINDA/EXM software with a mean of 0.0660±0.0550 mGy/MBq. Similarly, the d_rm_ calculated by Alan Selcuk et al. (24) using the same software was 0.1079±0.0319 mGy/MBq.

From these results, the reference phantom and S factor value greatly impacted the d_rm_. This is coherent with many studies and the difference in the dosimetry can be greater than 150% when using different phantoms and S factors (25,26,27,28). Hence, the selection of phantom is an important factor in the dosimetry calculation. Although the d_rm_ results showed some differences between both the software, the clinical outcome is still difficult to prove at this stage.

### Study Limitations

The small number of patients and the heterogeneity of the stages of the disease (local or distant metastasis) in the patient group might have biased the outcome of this study.

## Conclusion

The TIAC_WB_ calculated from a simplified excel spreadsheet was not statistically different from that of the software. The calculated d_rm_ using the simplified excel was non-inferior to that calculated by the software with an acceptable margin.

It can be concluded that a simplified excel spreadsheet can be used to calculate the d_rm_ in radioiodine therapy of DTC patients. The dose assessment using this method is feasible and relatively low cost compared to the commercial OLINDA/EXM image-based dosimetry software. Hence, the simplified Excel spreadsheet should increase the number of dosimetry studies in low or middle-income countries, though it requires further validation with more patients. Also, a method for improving TAC integration and the updated phantom for S-factor should be further considered.

## Figures and Tables

**Table 1:**
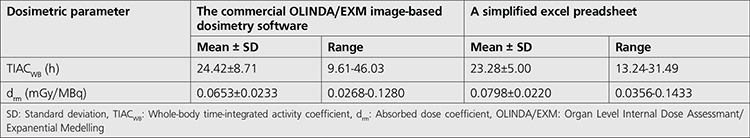
The mean (mean ± SD) and range (minimum - maximum) of the TIAC_WB_ in hours and d_rm_ in mGy/MBq calculated from the commercial OLINDA/EXM image-based dosimetry software and a simplified excel spreadsheet

**Table 2:**
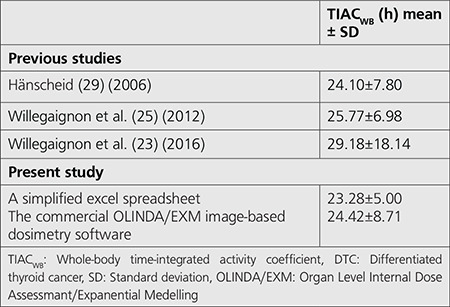
Comparison of the TIAC_WB_ of DTC patients in this study with previous studies

**Table 3:**
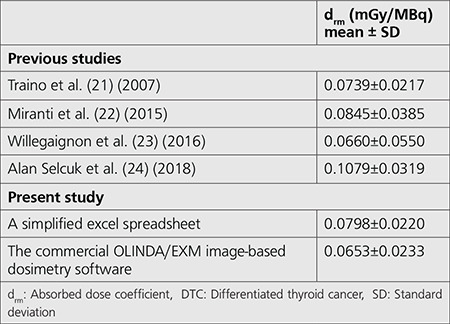
Comparison of the d_rm_ of DTC patients in this study with previous studies

## References

[1] Benua RS, Cicale NR, Sonenberg M, Rawson RW (1962). The relation of radioiodine dosimetry to results and complications in the treatment of metastatic thyroid cancer. Am J Roentgenol Radium Ther Nucl Med.

[2] Thomas SR, Samaratunga RC, Sperling M, Maxon HR (1993). Predictive estimate of blood dose from external counting data preceding radioiodine therapy for thyroid cancer. Nucl Med Biol.

[3] Lassmann M, Hanscheid H, Chiesa C, Hindorf C, Flux G, Luster M, EANM Dosimetry Committee (2008). EANM Dosimetry Committee series on standard operational procedures for pre-therapeutic dosimetry I: blood and bone marrow dosimetry in differentiated thyroid cancer therapy. Eur J Nucl Med Mol Imaging.

[4] Hindorf C, Glatting G, Chiesa C, Linden O, Flux G, Committee ED (2010). EANM Dosimetry Committee guidelines for bone marrow and whole-body dosimetry. Eur J Nucl Med Mol Imaging.

[5] Stabin MG, Siegel JA (2003). Physical models and dose factors for use in internal dose assessment. Health Phys.

[6] Hanscheid H, Lassmann M, Luster M, Kloos RT, Reiners C (2009). Blood dosimetry from a single measurement of the whole body radioiodine retention in patients with differentiated thyroid carcinoma. Endocr Relat Cancer.

[7] Sisson JC, Shulkin BL, Lawson S (2003). Increasing efficacy and safety of treatments of patients with well-differentiated thyroid carcinoma by measuring body retentions of 131I. J Nucl Med.

[8] Traino AC, Di Martino F, Boni G, Mariani G, Lazzeri M (2004). A minimally invasive method to evaluate 131I kinetics in blood. Radiat Prot Dosimetry.

[9] Sgouros G (1993). Bone marrow dosimetry for radioimmunotherapy: theoretical considerations. J Nucl Med.

[10] Stabin MG, Sparks RB, Crowe E (2005). OLINDA/EXM: the second-generation personal computer software for internal dose assessment in nuclear medicine. J Nucl Med.

[11] NCSS. Paired T-Test for Non-Inferiority. In: NCSS, editor. Utah K.

[12] Evans JD (1996.). Straightforward statistics for the behavioral sciences. Pacific Grove: Brooks/Cole Pub. Co.

[13] Bolch WE, Eckerman KF, Sgouros G, Thomas SR (2009). MIRD pamphlet No. 21: a generalized schema for radiopharmaceutical dosimetry--standardization of nomenclature. J Nucl Med.

[14] Gear JI, Cox MG, Gustafsson J, Gleisner KS, Murray I, Glatting G, Konijnenberg M, Flux GD (2018). EANM practical guidance on uncertainty analysis for molecular radiotherapy absorbed dose calculations. European Journal of Nuclear Medicine and Molecular Imaging.

[15] Götz TI, Schmidkonz C, Lang EW, Maier A, Kuwert T, Ritt P (2019). Factors affecting accuracy of S values and determination of time-integrated activity in clinical Lu-177 dosimetry. Ann Nucl Med.

[16] Pereira JM, Stabin MG, Lima FR, Guimaraes MI, Forrester JW (2010). Image quantification for radiation dose calculations--limitations and uncertainties. Health Phys.

[17] Hermanska J, Karny M, Zimak J, Jirsa L, Samal M, Vlcek P (2001). Improved prediction of therapeutic absorbed doses of radioiodine in the treatment of thyroid carcinoma. J Nucl Med.

[18] Jentzen W, Bockisch A, Ruhlmann M (2015). Assessment of Simplified Blood Dose Protocols for the Estimation of the Maximum Tolerable Activity in Thyroid Cancer Patients Undergoing Radioiodine Therapy Using 124I. J Nucl Med.

[19] Stabin MG, Cristy M, Ryman JC, Eckerman KF, Davis JL, Marshall D, Gehlen MK (1995.). Mathematical models and specific absorbed fractions of photon energy in the nonpregnant adult female and at the end of each trimester of pregnancy.

[20] NCSS. Two Correlated Proportions Non-Inferiority, Superiority, and Equivalence Tests. In: NCSS, editor. USA: Utah K.

[21] Traino AC, Ferrari M, Cremonesi M, Stabin MG (2007). Influence of total-body mass on the scaling of S-factors for patient-specific, blood-based redmarrow dosimetry. Phys Med Biol.

[22] Miranti A, Giostra A, Richetta E, Gino E, Pellerito RE, Stasi M (2015). Comparison of mathematical models for red marrow and blood absorbed dose estimation in the radioiodine treatment of advanced differentiated thyroid carcinoma. Phys Med Biol.

[23] Willegaignon J, Pelissoni RA, Lima BC, Sapienza MT, Coura-Filho GB, Queiroz MA, Buchpiguel CA (2016). Estimating (131)I biokinetics and radiation doses to the red marrow and whole body in thyroid cancer patients: probe detection versus image quantification. Radiol Bras.

[24] Alan Selcuk N, Toklu T, Beykan S, Karaaslan SI (2018). Evaluation of the dosimetry approaches in ablation treatment of thyroid cancer. J Appl Clin Med Phys.

[25] Willegaignon J, Sapienza MT, Buchpiguel CA (2012). Comparison of different dosimetric methods for red marrow absorbed dose calculation in thyroid cancer therapy. Radiat Prot Dosimetry.

[26] Josefsson A, Hobbs RF, Ranka S, Schwarz BC, Plyku D, Willegaignon de Amorim de Carvalho J, et al (2018). Comparative Dosimetry for (68) Ga-DOTATATE: Impact of Using Updated ICRP Phantoms, S Values, and Tissue-Weighting Factors. Journal of nuclear medicine : official publication. Soc Nucl Med.

[27] Lamart S, Bouville A, Simon SL, Eckerman KF, Melo D, Lee C (2011). Comparison of internal dosimetry factors for three classes of adult computational phantoms with emphasis on I-131 in the thyroid. Physics in medicine and biology..

[28] Andersson M, Johansson L, Minarik D, Leide-Svegborn S, Mattsson S (2014). Effective dose to adult patients from 338 radiopharmaceuticals estimated using ICRP biokinetic data, ICRP/ICRU computational reference phantoms and ICRP 2007 tissue weighting factors. EJNMMI physics..

[29] Hänscheid H, Lassmann M, Luster M, Thomas SR, Pacini F, Ceccarelli C, Ladenson PW, Wahl RL, Schlumberger M, Ricard M, Driedger A, Kloos T, Sherman AI, Haugen BR, Carriere V, Corone C, Reiners C (2006). Iodine biokinetics and dosimetry in radioiodine therapy of thyroid cancer: procedures and results of a prospective international controlled study of ablation after rhTSH or hormone withdrawal. J Nucl Med.

